# Hypoxia promotes the tolerogenic phenotype of plasmacytoid dendritic cells in head and neck squamous cell carcinoma

**DOI:** 10.1002/cam4.4511

**Published:** 2021-12-28

**Authors:** Cui Fan, Jichang Wu, Yilin Shen, Haixia Hu, Quan Wang, Yufeng Mao, Bin Ye, Mingliang Xiang

**Affiliations:** ^1^ Department of Otolaryngology & Head and Neck Surgery Ruijin Hospital Shanghai Jiao Tong University School of Medicine Shanghai China

**Keywords:** head and neck squamous cell carcinoma, hypoxia, immune suppression, plasmacytoid dendritic cells

## Abstract

**Objective:**

We aim to review the roles of plasmacytoid dendritic cells (pDCs) in head and neck squamous cell carcinoma (HNSCC) and explore the effects of hypoxia on the tolerogenic transformation of pDCs.

**Background:**

pDCs, best known as professional type I interferon‐secreting cells, play key roles in immune surveillance and antitumor immunity. Recently, pDCs have been shown to be tolerogenic and correlate with poor prognosis in a variety of cancers, including HNSCC. However, it remains unclear what drives the tolerogenic transformation of pDCs in the HNSCC microenvironment. Hypoxia, a prominent hallmark of the tumor microenvironment (TME) of HNSCC, can interfere with multiple immune cells and establish an immunosuppressive TME.

**Methods:**

In this review, we summarize the antitumor and protumor functions of pDCs, explore the effects of hypoxia on the migration and maturation of pDCs, and discuss related mechanisms in HNSCC.

**Conclusions:**

pDCs mainly display protumor functions in HNSCC. The hypoxic TME in HNSCC can enhance the migration of pDCs and inhibit the differentiation and maturation of pDCs, promoting the tolerogenic phenotype of pDCs.

## Graphical abstract

HNSCC is a type of immunogenic tumor characterized by the immunosuppressive tumor microenvironment. We review the antitumor and protumor functions of pDCs in HNSCC, discuss the effects of hypoxia on tumor‐infiltrating pDCs in HNSCC and recommend that hypoxia and related metabolites, such as lactate and adenosine, could damage the IFN‐α secretion and antigen presentation of pDCs, and enhance the migration and Treg induction of pDCs in HNSCC.

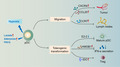

## 1 INTRODUCTION

Head and neck squamous cell carcinoma (HNSCC) may arise from the squamous cells of subsites such as oral cavity, nasal cavity, nasal sinuses, pharynx, larynx, or cervical esophagus.^1^ The 5‐year survival rate of HNSCC is rather poor due to high risks of local recurrence and distant metastasis.^2^ In recent years, the vital role of the tumor microenvironment (TME), especially the tumor immune microenvironment, has been increasingly appreciated in HNSCC. The host immune responses play a determinant role in the tumorigenesis and progression of HNSCC. It has been reported that recurrent HNSCC is characterized by an immunosuppressive TME with complex immune escape mechanisms.^3^ Moreover, tumor‐infiltrating lymphocytes, which are remodeled by the TME, have been proved to be independent prognostic factors in HNSCC.^4^, ^5^

As the most prominent feature of HNSCC, hypoxia facilitates tumor progression and correlates with recurrence, chemotherapy resistance, and poor survival. In the HNSCC TME, hypoxia plays central roles in the dysregulation of immune cells and the formation of an immunosuppressive TME by interfering with innate and adaptive immune responses.^6^, ^7^ The hypoxic TME drives the plasticity of immune cells via the hypoxia‐inducible factor (HIF) signaling pathway, such as enhancing M1 macrophage polarization and migration, promoting regulatory T cell (Treg) and T helper type 17 (Th17) cell differentiation, suppressing effector T‐cell functions, and upregulating pro‐inflammatory cytokine secretion by dendritic cells (DCs).^8^, ^9^ Plasmacytoid DCs (pDCs), a specialized DC subset, have traditionally been considered to contribute to immune surveillance and antitumor immunity owing to their powerful capacity to secrete type I interferon (IFN), especially IFN‐α, which is a crucial antitumor cytokine.^10^ However, the functions of tumor‐infiltrating pDCs have been controversial in recent years. It has been reported that abundant pDC infiltration in HNSCC,^11^ hepatocellular carcinoma,^12^ and gastric cancer^13^ was often correlated with poor prognosis. The antitumor functions of pDCs in these cancers are impaired under the influence of TME, making these cells favorable to tumor growth instead of executing immune surveillance. The mechanisms of the functional transformation of pDCs in HNSCC remain unclear. In this article, we comprehensively review the roles of pDCs in HNSCC, analyze the effects of the hypoxic TME on their functions, and discuss relevant mechanisms.

## 2 DUAL ROLES OF PDCS

pDCs are best known as professional IFN‐α‐producing cells, responsible for over 95% IFN‐α in peripheral blood.^14^ Besides, they are also called the Swiss army knife of the immune regulatory network due to their multiple functions, such as secreting cytokines, presenting antigens, exerting cytotoxicity, and inducing immune tolerance.^15^ Human bone marrow‐derived pDCs are Lin^−^ CD4^+^ CD45RA^+^ CD123^+^ BDCA2^+^ BDCA4^+^ cells with a unique oval plasma cell morphology.^16^ From the perspective of activation status, pDCs could be divided into immature and mature pDCs. Immature pDCs reside in lymphoid and mucosal tissues homeostatically with a small population, which mainly perform antigen‐sensing ability and become activated mature pDCs under stress conditions, such as viral infections and tumorigenesis. Mature pDCs with upregulated expression of markers such as CD80, CD83, and CD86 could present antigens to T cells and prime adaptive immunity.^10^ Additionally, there are some subtypes of pDCs being defined based on different markers. CD2^high^ pDCs are unique for expressing lysozyme with increased interleukin (IL)‐12 p40 secretion and CD80 expression, which are efficient in priming activation of naive T cells.^17^ CCR9^+^ pDCs are found to be tolerogenic with enhanced ability to induce Foxp3^+^ Treg.^18^ CD56^+^ pDCs with excellent antigen presenting and cytotoxic capacity could serve as killer pDCs.^19^ OX40^+^ pDCs exhibit an immunostimulatory feature with increased CD40, CD86, and CD80 expression.^20^ Upon pathogen stimulation through Toll‐like receptor 7/9 (TLR7/9), the myeloid differentiation factor 88 (MyD88)‐dependent downstream pathways, such as the interferon regulatory factor (IRF), NF‐κB, and MAPK pathways, are initiated within pDCs.^21^ On the one hand, activated pDCs with a mature dendritic morphology present antigens to T cells and prime adaptive immunity. On the other hand, mature pDCs are able to secrete a large amount of IFN‐α and other anti‐inflammatory factors, such as tumor necrosis factor (TNF)‐α and IL‐6.^22^

### 2.1 Antitumoral roles of pDCs

**FIGURE 1:**
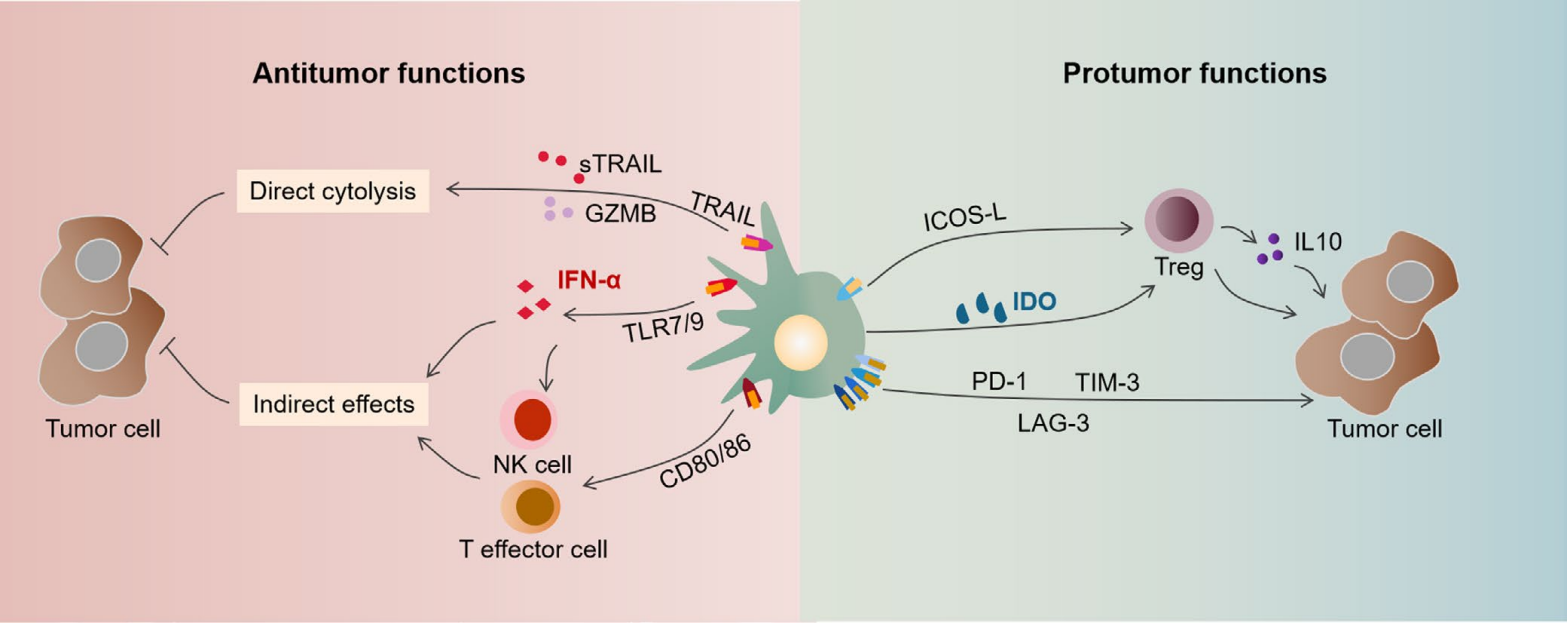
Antitumor and protumor functions of pDCs. Upon activation via TLR7/9, mature pDCs exhibit dendritic morphology and exert antitumor effects. The direct tumoricidal ability of pDCs mainly depends on the expression of TRAIL and GZMB. Indirectly, pDCs produce a large amount of IFN‐α, which induces NK cell activation. In addition, mature pDCs expressing MHC Class I, MHC Class II and markers, such as CD80 and CD86, can present antigens to T effector cells to mediate the killing of tumor cells. Tolerogenic pDCs exhibit plasma cell morphology and exert protumoral effects. With ICOSL and IDO expression, pDCs induce the differentiation and IL‐10 secretion of Tregs. Upregulated PD‐1, TIM‐3, and LAG‐3 expression of pDCs also favors tumor progression

As professional antigen‐presenting cells, mature pDCs undoubtedly have antitumoral properties. OX40^+^ pDCs were found to be immunostimulatory in HNSCC, facilitating antitumor immunity through IFN‐α secretion, CD8^+^ T‐cell activation, and cytolysis.^20^ pDCs can play both direct and indirect cytotoxic roles in antitumor immunity (Figure 1). Directly, pDCs contact with tumor cells and induce cytolysis via TNF‐related apoptosis‐inducing ligand (TRAIL)‐dependent pathway and the secretion of cytotoxic cytokines such as TNF‐α, granzyme B (GZMB), and soluble TRAIL.^19^ For instance, in skin malignancies such as melanoma and basal cell carcinoma, the TLR7 agonist imiquimod can enhance the cytotoxic function of pDCs, which mainly depended on TRAIL and GZMB secretion.^23^, ^24^ The indirect tumoricidal effects of pDCs are primarily mediated by IFN‐α secretion via the MyD88/IRF7 pathway.^25^ As a member of the interferon family, IFN‐α is capable of inducing tumor cell apoptosis and inhibiting tumor proliferation, vascularization, and metastasis.^26^ In addition, IFN‐α can also induce Th1 cell polarization and enhance natural killer (NK) cell and cytotoxic T lymphocyte (CTL) activity, and thereby, boosting antitumor immune response.^27^, ^28^ Moreover, pDCs have the ability to sensitively capture and present antigens to prime the T‐cell immune response by expressing major histocompatibility complex class II (MHC II) and they can also recruit and activate NK cells to exert cytotoxic effects.^29^ For these reasons, active pDCs in vitro and TLR7/9 agonists are applied in the immunotherapy of various cancers, such as breast cancer and melanoma, and have achieved encouraging outcomes.^30^, ^31^

### 2.2 Protumoral roles of pDCs

In recent years, the role of pDCs in tumor immune tolerance has become increasingly prominent. Tumor‐infiltrating pDCs often show an immature phenotype with defective abilities of IFN‐α production and antigen presentation, which facilitate tumor immune escape instead of immune surveillance (Figure 1). In oral squamous cell carcinoma (OSCC), increased infiltrating pDCs accompanied by reduced IFN‐α and IL‐6 secretion were found in tumor tissues compared to adjacent normal tissues, indicating defective functions of pDCs. Moreover, the degree of pDCs‐infiltrating OSCC correlated with lymph node metastasis and poor survival and could serve as an independent prognostic factor.^32^ Yang et al. reported that pDC depletion induced by antagonizing CD317 could enhance the antitumor immune response and inhibit tumor growth in a mouse model of HNSCC. Moreover, the researchers found that in human HNSCC, pDC infiltration was associated with immunosuppressive markers such as Foxp3, PD‐1, LAG‐3, and TIM‐3.^22^, ^33^ Additionally, pDCs‐infiltrating gastric cancer and breast cancer are found to support tumor progress by promoting Treg and Th2 cell differentiation and IL‐10, IL‐5, and IL‐13 secretion.^34^, ^35^

Tregs play an indispensable part in establishing immunosuppressive TME and promoting tumor immune escape in HNSCC.^36^ Tumor‐infiltrating pDCs are capable of inducing Treg differentiation and maturation, which are primarily mediated by the inducible costimulator ligand (ICOSL) and indoleamine 2,3‐dioxygenase (IDO) pathways. With high expression of ICOSL, tumor‐associated pDCs can recruit and activate ICOS^+^ Tregs and promote IL‐10 secretion.^37^ IDO, a crucial rate‐limiting enzyme in tryptophan metabolism, is highly expressed in human pDCs and is critical for maintaining the tolerogenic functions of pDCs. IDO was reported to be essentially involved in the pDC‐mediated induction of the differentiation of CD4^+^ CD25^−^ T cells into CD4^+^ CD25^+^ Foxp3^+^ Tregs. Moreover, the production of Tregs was inhibited by IDO blockers and could be restored by the addition of kynurenine.^38^, ^39^ Accumulated Tregs with high PD‐1 expression inhibit the proliferation of naive T cells and the cytotoxic ability of NK cells, leading to enhanced immunosuppression in HNSCC.^40^

## 3 THE INFLUENCE OF THE HYPOXIC TME ON THE FUNCTIONAL SWITCH IN PDCS IN HNSCC

**FIGURE 2:**
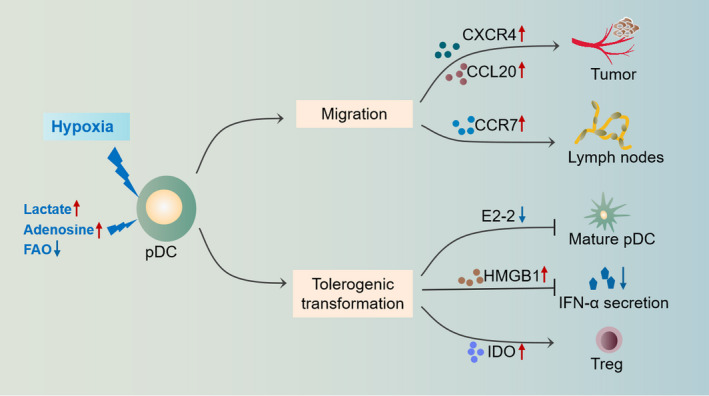
The influence of hypoxia and related metabolites on pDCs in HNSCC. A hypoxic TME promotes the recruitment of pDCs to tumor tissues via the upregulation of chemokines such as CXCR4 and CCL20 in a HIF‐1α‐dependent manner. The homing of pDCs to lymph nodes is also enhanced under hypoxic conditions due to upregulated CCR7. pDCs experience functional switching under hypoxic TME conditions. The maturation of pDCs is suppressed due to HIF‐1α‐mediated E2‐2 inhibition. Increased HMGB1 secretion by tumor cells inhibits the IFN‐α production of pDCs. Upregulated expression of IDO enhances pDC‐mediated induction of Tregs and hampers their antigen presentation ability

In HNSCC, tumor‐associated pDCs experience functional switch, thus promoting tumor growth and invasion. The mechanisms behind this switch have not been fully elucidated. Due to the rapid growth of solid tumors, the tumor core and infiltrative margin areas are insufficiently oxygenated.^41^ Hypoxia is the central determinant of an immunosuppressive TME through modulating tumor‐associated immune checkpoints and enhancing immunosuppressive functions of multiple immune cell types, which closely correlate with tumor aggressiveness and clinical prognosis.^6^ Here, we mainly discuss the potential effects of hypoxia and its related metabolites on the migration and function of pDCs and analyze the underlying mechanisms (Figure 2).

### 3.1 Hypoxia enhances the migration of pDCs

Cellular metabolism, especially glycolysis and oxidative phosphorylation, has been proven to influence the migration of various immune cells. Hypoxia, which promotes the aerobic glycolysis of tumor cells, contributes to DC cell migration.^42^ Immune cell migration is directed by changes in related chemokines. For pDCs, the most critical chemokines are CXC receptor type 4 (CXCR4) and chemokine receptor 7 (CCR7).^21^ pDC migration is enhanced under hypoxic conditions, mainly due to the modulation of related chemokines. CXCR4 and its ligand chemokine stromal derived factor‐1 (SDF‐1) play crucial roles in the recruitment of pDCs from peripheral blood to tumor and surrounding tissues.^43^ The SDF‐1/CXCR4 pathway is well known to be involved in tumor progression. In HNSCC, high expression of SDF‐1/CXCR4 indicates invasive tumor behavior and correlates with local proliferation and lymphatic and distant metastasis.^44^ De‐Colle et al. noted that high expression of SDF‐1/CXCR4 was an independent prognostic factor for poor survival in locally advanced HNSCC during initial radiotherapy and chemotherapy.^45^ In breast cancer, pDCs were reported to contribute to lymph node metastasis through SDF‐1/CXCR4 pathway.^46^ Hypoxia has been reported to be a vital priming condition for CXCR4 expression through HIF‐1α‐dependent pathways.^47^ Transcriptome analysis indicated significantly elevated CXCR4 expression in immature DCs under hypoxic condition.^48^ Ishikawa et al. found that there was a positive correlation between HIF‐1α and CXCR4 expression by immunohistochemical analysis of 85 cases of OSCC samples, and further in vitro experiments proved that HIF‐1α inhibition could reduce the expression of CXCR4.^49^ These evidences suggest that in the HNSCC TME, hypoxia might promote the migration of pDCs into tumor tissues through the HIF‐1α/SDF‐1/CXCR4 pathway.

CCR7, which binds to C–C chemokine ligand 19/21 (CCL19/21), is an essential promoter for the homing of pDCs to lymph nodes.^50^ In HNSCC, hypoxia was reported to be a crucial inducing factor for the upregulation of CCR7 expression, which correlated with lymphatic metastasis.^51^ The hypoxia‐induced glycolysis could promote the oligomerization of CCR7 in a HIF‐1α‐dependent way.^52^ Moreover, restraining HIF‐1α‐dependent glycolysis with a long non‐coding RNA lnc‐Dpf3 efficiently prevents DC migration mediated by CCR7.^53^ The hypoxic TME in HNSCC may promote the migration of pDCs to tumor‐draining lymph nodes by upregulating CCR7 expression, facilitating lymphatic metastasis. In addition, Bosco et al. explored the transcriptome of human monocytes under hypoxic conditions and identified marked upregulation of CCL20 induced by hypoxia.^54^ CCL20 is capable of attracting immature pDCs into tumor tissues, which tend to induce Treg differentiation and contribute to a tolerant TME.^55^

### 3.2 Hypoxia reprograms the immune functions of pDCs

**FIGURE 3:**
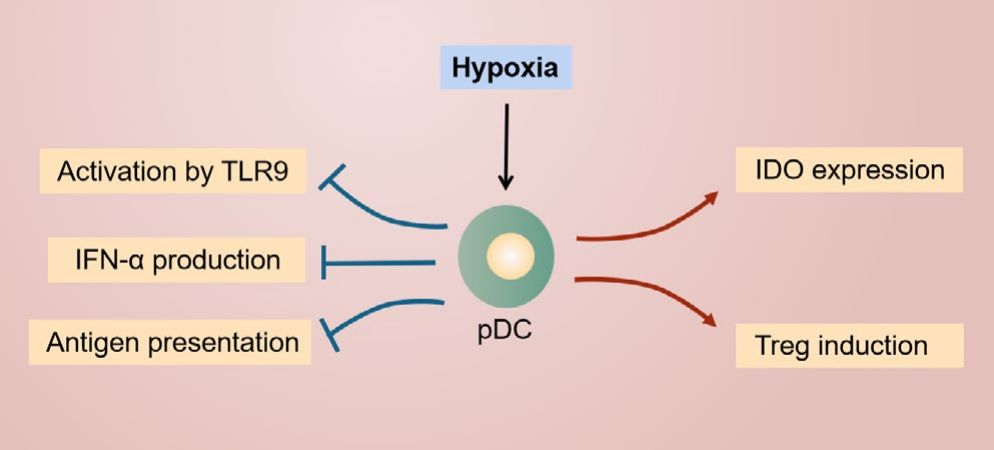
Hypoxia reprograms the immune functions of pDCs. The activation of pDCs in response to TLR9 stimulation is suppressed under hypoxic conditions, accompanied by impaired abilities of IFN‐α production and antigen presentation. Instead, hypoxia promotes the tolerogenic functions of pDCs, mainly including upregulated IDO expression and Treg induction

Mature pDCs can secrete IFN‐α and exhibit antitumor effects, whereas tumor‐associated pDCs with immature phenotypes show tolerogenic properties related to the hypoxic TME (Figure 3). Hypoxia is a negative regulator for pDCs.^56^ Yang et al. compared the differences in the gene profiles of DCs under normal and hypoxic conditions and found that the expression of genes related to T‐cell stimulation in DCs exposed to hypoxia was markedly downregulated, suggesting the diminished antigen presentation ability of pDCs.^57^ They further found that under hypoxic condition, DCs cultured in vitro exhibited defective abilities of activation, migration, antigen capture, and phagocytosis and were more inclined to induce Th2 differentiation of naive CD4^+^ T cells, which promoted immunosuppression.^58^

The transcription factor E2‐2 modulates the differentiation and maturation of pDCs in a STAT3‐dependent manner, and dysregulation of this process could result in the spontaneous differentiation of mature pDCs into classic DCs.^59^ Weigert et al. reported that the numbers of pDCs in the blood, spleen, and bone marrow were increased in HIF‐1α knockout mice and proposed that under hypoxic condition, HIF‐1α could induce the expression of inhibitor of DNA binding 2 (ID2), which inhibits E2‐2 and leads to disrupted differentiation and maturation of pDCs.^56^ In a recent research, elevated ID2 expression was found in HNSCC and closely correlated with highly aggressive tumors.^60^ These findings suggest that the HIF‐1α/ID2/E2‐2 pathway may be essential in hypoxia‐mediated regulation of the differentiation and maturation of pDCs. pDCs‐infiltrating HNSCC have been found to be less efficient in response to the stimulation of CpG motif with inhibited IFN‐α secretion and the dysfunction of these cells was partly due to downregulation of TLR9.^11^ High mobility group box 1 protein (HMGB1), a damage‐associated molecular pattern protein, is upregulated in hypoxic tumor tissues, which promotes tumor invasion and correlates with poor prognosis in HNSCC.^61^ HMGB1 inhibits TLR9‐mediated pDC maturation and cytokine secretion, such as IFN‐α, IL‐6, IL‐12, TNF‐α, and inducible protein 10 (IP‐10), promoting tolerogenic alterations in pDCs.^62^, ^63^ Downregulation of IP‐10 also suppresses the recruitment of Th1 and NK cells. Moreover, it has been reported that the impaired functions of pDCs‐infiltrating cervical cancer could be restored through HMGB1 inhibition.^55^ Based on these results, we hypothesize that HMGB1 might be involved in inducing tolerogenic pDCs and immune escape in the hypoxic TME of HNSCC.

As an important immune checkpoint in HNSCC, IDO plays a critical role in the induction of Treg differentiation by pDCs.^64^ IDO, the marker of long‐term tolerance in DCs, is upregulated under hypoxic conditions and can promote tolerogenic functions of DCs by modulating the metabolism of tryptophan.^65^ In HNSCC, IDO was proposed to contribute to tumor resistance to anti‐PD‐1 immunotherapy.^66^ In addition, the serum kynurenine/tryptophan (kyn/trp) ratio, the marker of IDO activity, could serve as a prognostic factor in HNSCC immunotherapy.^67^ Ye et al. found that hypoxia upregulated the level of IDO expression in hepatocellular carcinoma via the HIF‐1α/CCL20/STAT1/IDO pathway, contributing to immune tolerance and tumor metastasis.^68^ These evidences indicate that hypoxia can regulate the tolerogenic functions of pDCs through IDO regulation. Yamahira et al. treated the leukemia plasmacytoid dendritic cell line PMDC05 with Toho‐1, a novel IDO inhibitor, and found that Toho‐1 enhanced the abilities of pDCs to present antigens and induce CTLs and could be applied in cancer immunotherapy.^69^

### 3.3 The effects of hypoxic metabolites on pDCs

Hypoxic TME changes the way of tumor energy metabolism and makes glycolysis a priority for the energy supply of tumor cells, leading to lactate and adenosine accumulation in the hypoxic TME.^70^ Accumulated lactate and an acid TME impairs normal functions of immune cells and weakens antitumor immunity, thus facilitating tumor immune escape.^71^ In a prospective study on HNSCC after radiation, high levels of lactate were correlated with poor overall survival and high risks of recurrence.^72^ Lactate accumulation in the TME could hamper the T‐cell activation and IFN‐α secretion by DCs in lung cancer, which was partly due to TLR3 and stimulator of interferon genes (STING) inhibition.^73^ Lactate can promote pDC reprogramming. On the one hand, lactate inhibits IFN‐α secretion of tumor‐infiltrating pDCs by binding to the GPR81 receptor on the surface of pDCs, resulting in disturbed calcium transport and glycolysis. On the other hand, lactate enhances the catabolism of tryptophan and the production of kynurenine, promoting Treg induction of pDCs.^74^

The accumulation of extracellular adenosine has also been reported to contribute to the antitumor immune response in the hypoxic TME, which partly depends on interrupting the antigen presentation ability of DCs.^75^ Extracellular adenosine can not only promote the recruitment of immature pDCs to inflammatory tissues through the A1 receptor but also inhibit the ability of pDCs to secrete cytokines such as IFN‐α and IL‐12 through the A2a receptor, limiting the degree of immunogenic response.^76^ Ma et al. reported that the expression levels of A2a in HNSCC were related to tumor volume, lymph node metastasis, recurrence, and preoperative adjuvant chemotherapy, and that the application of A2a receptor antagonists could enhance the antitumor effects of CD8^+^ T cells and reduce Treg infiltration, thus markedly impeding tumor growth.^77^ Aside from glycolysis, hypoxia also affects the progress of fatty acid β‐oxidation (FAO), which is crucial for pDC activation and maturation. The FAO inhibition and fatty acid accumulation impedes full activation of pDCs in an ATP‐dependent manner.^78^ In the hypoxic TME, HIF‐1α‐mediated FAO inhibition facilitates tumor progression.^79^ The overexpression of FAO‐related enzymes was also reported to show protective effects against tumor in HNSCC.^80^ In summary, lactate and adenosine accumulation and FAO suppression in the hypoxic TME of HNSCC might accelerate tumor growth by promoting pDC reprogramming.

## 4 CONCLUSIONS AND PERSPECTIVES

HNSCC is a highly aggressive malignant tumor with an immunosuppressive TME. The disorder of immune system is believed to critically contribute to the tumorigenesis and progression of HNSCC. Hypoxia, a key feature of HNSCC, drives the establishment of an immunosuppressive TME and correlates with poor prognosis and chemotherapy tolerance in HNSCC. As professional IFN‐α‐producing cells, pDCs are distinguished by their multiple functions, especially their tolerogenic ability, which is the primary function of pDCs in HNSCC immunity. In this article, we reviewed the antitumor and protumor functions of pDCs and discussed the influences of hypoxia on pDCs in HNSCC. A hypoxic TME can not only enhance the migration of pDCs but also inhibit the differentiation and maturation of pDCs, promoting tolerogenic pDCs with defective IFN‐α secretion and antigen presentation abilities and enhanced Treg induction abilities. Since there are few researches about hypoxia and pDCs in HNSCC, the detailed mechanisms between them remain largely unknown. Do the effects of hypoxia on pDCs occur in the early stage of HNSCC? Which is the prominent subtype of pDC‐infiltrating HNSCC? What is the key mechanism in hypoxia‐mediated modulation of pDCs and how can the antitumoral ability of pDCs be restored? Will tolerogenic pDCs accelerate the degree of hypoxia in the HNSCC TME? A better understanding of pDCs in the hypoxic TME could broaden our exploration into tumor immunosuppression and promote the manipulation of hypoxic stress and pDC activation in immunotherapy for HNSCC.

## CONFLICT OF INTEREST

The authors declare that there are no known competing financial interests or personal relationships that could influence their work.

## AUTHOR CONTRIBUTIONS

(I) Conception and design: C F, ML X, and B Y; (II) Administrative support: ML X; (III) Provision of study materials or patients: YL S, HX H, and Q W; (IV) Collection and assembly of data: JC W, B Y, and YF M; (V) Data analysis and interpretation: C F, JC W, and YL S; (VI) Manuscript writing: All authors; (VII) Final approval of manuscript: All authors.

## ETHICAL STATEMENT

The authors are accountable for all aspects of the work in ensuring that questions related to the accuracy or integrity of any part of the work are appropriately investigated and resolved.

## Data Availability

All the information provided in the article are available.

## References

[1] Marur S , Forastiere A . Head and neck squamous cell carcinoma: update on epidemiology, diagnosis, and treatment. Mayo Clin Proc. 2016;91:386‐396. 26944243 10.1016/j.mayocp.2015.12.017

[2] Siegel R , Miller K , Jemal A . Cancer statistics, 2019. CA Cancer J Clin. 2019;69:7‐34. 30620402 10.3322/caac.21551

[3] Moy J , Moskovitz J , Ferris R . Biological mechanisms of immune escape and implications for immunotherapy in head and neck squamous cell carcinoma. Eur J Cancer. 2017;76:152‐166. 28324750 10.1016/j.ejca.2016.12.035 PMC5459368

[4] Borsetto D , Tomasoni M , Payne K , et al. Prognostic significance of CD4^+^ and CD8^+^ tumor‐infiltrating lymphocytes in head and neck squamous cell carcinoma: a meta‐analysis. Cancers (Basel). 2021;13:781. 33668519 10.3390/cancers13040781 PMC7918220

[5] Chen SMY , Krinsky AL , Woolaver RA , Wang X , Chen Z , Wang JH . Tumor immune microenvironment in head and neck cancers. Mol Carcinog. 2020;59:766‐774. 32017286 10.1002/mc.23162 PMC7282929

[6] Wang B , Zhao Q , Zhang Y , Liu Z , Jiang X . Targeting hypoxia in the tumor microenvironment: a potential strategy to improve cancer immunotherapy. J Exp Clin Cancer Res. 2021;40:24. 33422072 10.1186/s13046-020-01820-7 PMC7796640

[7] Noman M , Hasmim M , Messai Y , et al. Hypoxia: a key player in antitumor immune response. A review in the theme: cellular responses to hypoxia. Am J Physiol Cell Physiol. 2015;309:C569‐579. 26310815 10.1152/ajpcell.00207.2015 PMC4628936

[8] Chouaib S , Messai Y , Couve S , Escudier B , Hasmim M , Noman M . Hypoxia promotes tumor growth in linking angiogenesis to immune escape. Front Immunol. 2012;3:21. 22566905 10.3389/fimmu.2012.00021 PMC3341970

[9] Colgan S , Furuta G , Taylor C . Hypoxia and innate immunity: keeping up with the HIFsters. Annu Rev Immunol. 2020;38:341‐363. 31961750 10.1146/annurev-immunol-100819-121537 PMC7924528

[10] Reizis B . Plasmacytoid dendritic cells: development, regulation, and function. Immunity. 2019;50:37‐50. 30650380 10.1016/j.immuni.2018.12.027 PMC6342491

[11] Hartmann E , Wollenberg B , Rothenfusser S , et al. Identification and functional analysis of tumor‐infiltrating plasmacytoid dendritic cells in head and neck cancer. Cancer Res. 2003;63:6478‐6487. 14559840

[12] Zhou Z , Xin H , Li J , Hu Z , Luo C , Zhou S . Intratumoral plasmacytoid dendritic cells as a poor prognostic factor for hepatocellular carcinoma following curative resection. Cancer Immunol Immunother. 2019;68:1223‐1233. 31201473 10.1007/s00262-019-02355-3 PMC11028119

[13] Liu X , Yu H , Yan C , et al. Plasmacytoid dendritic cells and ICOS regulatory T cells predict poor prognosis in gastric cancer: a pilot study. J Cancer. 2019;10:6711‐6715. 31777600 10.7150/jca.34826 PMC6856898

[14] Siegal F , Kadowaki N , Shodell M , et al. The nature of the principal type 1 interferon‐producing cells in human blood. Science. 1999;284(5421):1835‐1837. 10364556 10.1126/science.284.5421.1835

[15] Karrich J , Jachimowski L , Uittenbogaart C , Blom B . The plasmacytoid dendritic cell as the Swiss army knife of the immune system: molecular regulation of its multifaceted functions. J Immunol. 1950;2014(193):5772‐5778. 10.4049/jimmunol.1401541 PMC4258904 25480956

[16] Merad M , Manz M . Dendritic cell homeostasis. Blood. 2009;113:3418‐3427. 19176316 10.1182/blood-2008-12-180646 PMC2668851

[17] Matsui T , Connolly JE , Michnevitz M , et al. CD2 distinguishes two subsets of human plasmacytoid dendritic cells with distinct phenotype and functions. J Immunol. 2009;182(11):6815‐6823. 19454677 10.4049/jimmunol.0802008 PMC2749454

[18] Hadeiba H , Sato T , Habtezion A , Oderup C , Pan J , Butcher E . CCR9 expression defines tolerogenic plasmacytoid dendritic cells able to suppress acute graft‐versus‐host disease. Nat Immunol. 2008;9(11):1253‐1260. 18836452 10.1038/ni.1658 PMC2901237

[19] Tel J , Smits E , Anguille S , Joshi R , Figdor C , de Vries I . Human plasmacytoid dendritic cells are equipped with antigen‐presenting and tumoricidal capacities. Blood. 2012;120:3936‐3944. 22966165 10.1182/blood-2012-06-435941

[20] Poropatich K , Dominguez D , Chan W , et al. OX40+ plasmacytoid dendritic cells in the tumor microenvironment promote antitumor immunity. J Clin Investig. 2020;130:3528‐3542. 32182225 10.1172/JCI131992 PMC7324178

[21] Alculumbre S , Raieli S , Hoffmann C , Chelbi R , Danlos F , Soumelis V . Plasmacytoid pre‐dendritic cells (pDC): from molecular pathways to function and disease association. Semin Cell Dev Biol. 2019;86:24‐35. 29444460 10.1016/j.semcdb.2018.02.014

[22] Mitchell D , Chintala S , Dey M . Plasmacytoid dendritic cell in immunity and cancer. J Neuroimmunol. 2018;322:63‐73. 30049538 10.1016/j.jneuroim.2018.06.012

[23] Kalb M , Glaser A , Stary G , Koszik F , Stingl G . TRAIL(+) human plasmacytoid dendritic cells kill tumor cells in vitro: mechanisms of imiquimod‐ and IFN‐α‐mediated antitumor reactivity. J Immunol. 1950;2012(188):1583‐1591. 10.4049/jimmunol.1102437 22231699

[24] Stary G , Bangert C , Tauber M , Strohal R , Kopp T , Stingl G . Tumoricidal activity of TLR7/8‐activated inflammatory dendritic cells. J Exp Med. 2007;204:1441‐1451. 17535975 10.1084/jem.20070021 PMC2118597

[25] Tel J , Anguille S , Waterborg C , Smits E , Figdor C , de Vries I . Tumoricidal activity of human dendritic cells. Trends Immunol. 2014;35:38‐46. 24262387 10.1016/j.it.2013.10.007 PMC7106406

[26] Belardelli F , Ferrantini M , Proietti E , Kirkwood J . Interferon‐alpha in tumor immunity and immunotherapy. Cytokine Growth Factor Rev. 2002;13:119‐134. 11900988 10.1016/s1359-6101(01)00022-3

[27] Fenton S , Saleiro D , Platanias L . Type I and II interferons in the anti‐tumor immune response. Cancers (Basel). 2021;13:1037. 33801234 10.3390/cancers13051037 PMC7957896

[28] Müller L , Aigner P , Stoiber D . Type I interferons and natural killer cell regulation in cancer. Front Immunol. 2017;8:304. 28408907 10.3389/fimmu.2017.00304 PMC5374157

[29] Koucký V , Bouček J , Fialová A . Immunology of plasmacytoid dendritic cells in solid tumors. A brief review. Cancers (Basel). 2019;11:470. 10.3390/cancers11040470 PMC6520684 30987228

[30] Charles J , Chaperot L , Hannani D , et al. An innovative plasmacytoid dendritic cell line‐based cancer vaccine primes and expands antitumor T‐cells in melanoma patients in a first‐in‐human trial. Oncoimmunology. 2020;9:1738812. 32313721 10.1080/2162402X.2020.1738812 PMC7153838

[31] Adams S , Kozhaya L , Martiniuk F , et al. Topical TLR7 agonist imiquimod can induce immune‐mediated rejection of skin metastases in patients with breast cancer. Clin Cancer Res. 2012;18:6748‐6757. 22767669 10.1158/1078-0432.CCR-12-1149 PMC3580198

[32] Han N , Zhang Z , Liu S , et al. Increased tumor‐infiltrating plasmacytoid dendritic cells predicts poor prognosis in oral squamous cell carcinoma. Arch Oral Biol. 2017;78:129‐134. 28242507 10.1016/j.archoralbio.2017.02.012

[33] Yang L , Mao L , Wu H , et al. pDC depletion induced by CD317 blockade drives the antitumor immune response in head and neck squamous cell carcinoma. Oral Oncol. 2019;96:131‐139. 31422204 10.1016/j.oraloncology.2019.07.019

[34] Sawant A , Hensel JA , Chanda D , et al. Depletion of plasmacytoid dendritic cells inhibits tumor growth and prevents bone metastasis of breast cancer cells. J Immunol. 2012;189(9):4258‐4265. 23018462 10.4049/jimmunol.1101855 PMC3531993

[35] Huang XM , Liu XS , Lin XK , et al. Role of plasmacytoid dendritic cells and inducible costimulator‐positive regulatory T cells in the immunosuppression microenvironment of gastric cancer. Cancer Sci. 2014;105(2):150‐158. 24261990 10.1111/cas.12327 PMC4317822

[36] Strauss L , Bergmann C , Gooding W , Johnson J , Whiteside T . The frequency and suppressor function of CD4+CD25highFoxp3+ T cells in the circulation of patients with squamous cell carcinoma of the head and neck. Clin Cancer Res. 2007;13:6301‐6311. 17975141 10.1158/1078-0432.CCR-07-1403

[37] Ito T , Yang M , Wang Y , et al. Plasmacytoid dendritic cells prime IL‐10‐producing T regulatory cells by inducible costimulator ligand. J Exp Med. 2007;204:105‐115. 17200410 10.1084/jem.20061660 PMC2118437

[38] Chen W , Liang X , Peterson A , Munn D , Blazar B . The indoleamine 2,3‐dioxygenase pathway is essential for human plasmacytoid dendritic cell‐induced adaptive T regulatory cell generation. J Immunol. 1950;2008(181):5396‐5404. 10.4049/jimmunol.181.8.5396 PMC2614675 18832696

[39] Fallarino F , Gizzi S , Mosci P , Grohmann U , Puccetti P . Tryptophan catabolism in IDO+ plasmacytoid dendritic cells. Curr Drug Metab. 2007;8(3):209‐216. 17430109 10.2174/138920007780362581

[40] Economopoulou P , Agelaki S , Perisanidis C , Giotakis E , Psyrri A . The promise of immunotherapy in head and neck squamous cell carcinoma. Ann Oncol. 2016;27:1675‐1685. 27380958 10.1093/annonc/mdw226

[41] Harris A . Hypoxia–a key regulatory factor in tumour growth. Nat Rev Cancer. 2002;2:38‐47. 11902584 10.1038/nrc704

[42] Khler T , Reizis B , Johnson RS , Weighardt H , Frster I . Influence of hypoxia‐inducible factor 1α on dendritic cell differentiation and migration. Eur J Immunol. 2012;42. 10.1002/eji.201142053 PMC6592818 22539295

[43] Vanbervliet B , Bendriss‐Vermare N , Massacrier C , et al. The inducible CXCR3 ligands control plasmacytoid dendritic cell responsiveness to the constitutive chemokine stromal cell‐derived factor 1 (SDF‐1)/CXCL12. J Exp Med. 2003;198:823‐830. 12953097 10.1084/jem.20020437 PMC2194187

[44] León X , Diez S , García J , et al. Expression of the CXCL12/CXCR4 chemokine axis predicts regional control in head and neck squamous cell carcinoma. Eur Arch Otorhinolaryngol. 2016;273:4525‐4533. 27328961 10.1007/s00405-016-4144-9

[45] De‐Colle C , Menegakis A , Mönnich D , et al. SDF‐1/CXCR4 expression is an independent negative prognostic biomarker in patients with head and neck cancer after primary radiochemotherapy. Radiother Oncol. 2018;126:125‐131. 29061496 10.1016/j.radonc.2017.10.008

[46] Gadalla R , Hassan H , Ibrahim S , et al. Tumor microenvironmental plasmacytoid dendritic cells contribute to breast cancer lymph node metastasis via CXCR4/SDF‐1 axis. Breast Cancer Res Treat. 2019;174(3):679‐691. 30632021 10.1007/s10549-019-05129-8

[47] Guan G , Zhang Y , Lu Y , et al. The HIF‐1α/CXCR4 pathway supports hypoxia‐induced metastasis of human osteosarcoma cells. Cancer Lett. 2015;357:254‐264. 25444927 10.1016/j.canlet.2014.11.034

[48] Ricciardi A , Elia AR , Cappello P , et al. Transcriptome of hypoxic immature dendritic cells: modulation of chemokine/receptor expression. Mol Cancer Res. 2008;6(2):175‐185. 18314479 10.1158/1541-7786.MCR-07-0391

[49] Ishikawa T , Nakashiro K , Klosek S , et al. Hypoxia enhances CXCR4 expression by activating HIF‐1 in oral squamous cell carcinoma. Oncol Rep. 2009;21:707‐712. 19212630

[50] Seth S , Oberdörfer L , Hyde R , et al. CCR7 essentially contributes to the homing of plasmacytoid dendritic cells to lymph nodes under steady‐state as well as inflammatory conditions. J Immunol. 1950;2011(186):3364‐3372. 10.4049/jimmunol.1002598 21296980

[51] Liu F , Safdar J , Li Z , et al. CCR7 regulates cell migration and invasion through MAPKs in metastatic squamous cell carcinoma of head and neck. Int J Oncol. 2014;45:2502‐2510. 25270024 10.3892/ijo.2014.2674

[52] Guak H , Al Habyan S , Ma E , et al. Glycolytic metabolism is essential for CCR7 oligomerization and dendritic cell migration. Nat Commun. 2018;9(1):2463. 29941886 10.1038/s41467-018-04804-6 PMC6018630

[53] Liu J , Zhang X , Chen K , et al. CCR7 chemokine receptor‐inducible lnc‐Dpf3 restrains dendritic cell migration by inhibiting HIF‐1α‐mediated glycolysis. Immunity. 2019;50(3):600‐615.e15. 30824325 10.1016/j.immuni.2019.01.021

[54] Bosco M , Puppo M , Santangelo C , et al. Hypoxia modifies the transcriptome of primary human monocytes: modulation of novel immune‐related genes and identification of CC‐chemokine ligand 20 as a new hypoxia‐inducible gene. J Immunol. 1950;2006(177):1941‐1955. 10.4049/jimmunol.177.3.1941 16849508

[55] Schutyser E , Struyf S , Van Damme J . The CC chemokine CCL20 and its receptor CCR6. Cytokine Growth Factor Rev. 2003;14:409‐426. 12948524 10.1016/s1359-6101(03)00049-2

[56] Weigert A , Weichand B , Sekar D , et al. HIF‐1α is a negative regulator of plasmacytoid DC development in vitro and in vivo. Blood. 2012;120:3001‐3006. 22936665 10.1182/blood-2012-03-417022

[57] Yang M , Ma C , Liu S , et al. Hypoxia skews dendritic cells to a T helper type 2‐stimulating phenotype and promotes tumour cell migration by dendritic cell‐derived osteopontin. Immunology. 2009;128:e237‐249. 19740309 10.1111/j.1365-2567.2008.02954.x PMC2753916

[58] Yang M , Liu Y , Ren G , et al. Increased expression of surface CD44 in hypoxia‐DCs skews helper T cells toward a Th2 polarization. Sci Rep. 2015;5:13674. 26323509 10.1038/srep13674 PMC4555176

[59] Ghosh H , Cisse B , Bunin A , Lewis K , Reizis B . Continuous expression of the transcription factor e2–2 maintains the cell fate of mature plasmacytoid dendritic cells. Immunity. 2010;33:905‐916. 21145760 10.1016/j.immuni.2010.11.023 PMC3010277

[60] Bae WJ , Koo BS , Lee SH , et al. Inhibitor of DNA binding 2 is a novel therapeutic target for stemness of head and neck squamous cell carcinoma. Br J Cancer. 2017;117(12):1810‐1818. 29096401 10.1038/bjc.2017.373 PMC5729481

[61] Liu Y , Xie C , Zhang X , et al. Elevated expression of HMGB1 in squamous‐cell carcinoma of the head and neck and its clinical significance. Eur J Cancer. 2010;46:3007‐3015. 20724142 10.1016/j.ejca.2010.07.016

[62] Demoulin S , Herfs M , Somja J , Roncarati P , Delvenne P , Hubert P . HMGB1 secretion during cervical carcinogenesis promotes the acquisition of a tolerogenic functionality by plasmacytoid dendritic cells. Int J Cancer. 2015;137:345‐358. 25492101 10.1002/ijc.29389

[63] Liu Y , Yan W , Tohme S , et al. Hypoxia induced HMGB1 and mitochondrial DNA interactions mediate tumor growth in hepatocellular carcinoma through Toll‐like receptor 9. J Hepatol. 2015;63:114‐121. 25681553 10.1016/j.jhep.2015.02.009 PMC4475488

[64] Yun T , Lee J , Machmach K , et al. Indoleamine 2,3‐dioxygenase‐expressing aortic plasmacytoid dendritic cells protect against atherosclerosis by induction of regulatory T cells. Cell Metab. 2016;23:852‐866. 27166946 10.1016/j.cmet.2016.04.010

[65] Mellor A , Lemos H , Huang L . Indoleamine 2,3‐dioxygenase and tolerance: where are we now? Front Immunol. 2017;8:1360. 29163470 10.3389/fimmu.2017.01360 PMC5663846

[66] Holmgaard R , Zamarin D , Munn D , Wolchok J , Allison J . Indoleamine 2,3‐dioxygenase is a critical resistance mechanism in antitumor T cell immunotherapy targeting CTLA‐4. J Exp Med. 2013;210:1389‐1402. 23752227 10.1084/jem.20130066 PMC3698523

[67] Botticelli A , Mezi S , Pomati G , et al. Tryptophan catabolism as immune mechanism of primary resistance to anti‐PD‐1. Front Immunol. 2020;11:1243. 32733441 10.3389/fimmu.2020.01243 PMC7358280

[68] Ye L , Chen W , Bai X , et al. Hypoxia‐induced epithelial‐to‐mesenchymal transition in hepatocellular carcinoma induces an immunosuppressive tumor microenvironment to promote metastasis. Cancer Res. 2016;76:818‐830. 26837767 10.1158/0008-5472.CAN-15-0977

[69] Yamahira A , Narita M , Iwabuchi M , et al. Activation of the leukemia plasmacytoid dendritic cell line PMDC05 by Toho‐1, a novel IDO inhibitor. Anticancer Res. 2014;34:4021‐4028. 25075025

[70] Vaupel P , Multhoff G . Accomplices of the hypoxic tumor microenvironment compromising antitumor immunity: adenosine, lactate, acidosis, vascular endothelial growth factor, potassium ions, and phosphatidylserine. Front Immunol. 2017;8:1887. 29312351 10.3389/fimmu.2017.01887 PMC5742577

[71] Ippolito L , Morandi A , Giannoni E , Chiarugi P . Lactate: a metabolic driver in the tumour landscape. Trends Biochem Sci. 2019;44:153‐166. 30473428 10.1016/j.tibs.2018.10.011

[72] Blatt S , Voelxen N , Sagheb K , et al. Lactate as a predictive marker for tumor recurrence in patients with head and neck squamous cell carcinoma (HNSCC) post radiation: a prospective study over 15 years. Clin Oral Investig. 2016;20:2097‐2104. 10.1007/s00784-015-1699-6 26728026

[73] Caronni N , Simoncello F , Stafetta F , et al. Downregulation of membrane trafficking proteins and lactate conditioning determine loss of dendritic cell function in lung cancer. Cancer Res. 2018;78:1685‐1699. 29363545 10.1158/0008-5472.CAN-17-1307

[74] Raychaudhuri D , Bhattacharya R , Sinha B , et al. Lactate induces pro‐tumor reprogramming in intratumoral plasmacytoid dendritic cells. Front Immunol. 2019;10:1878. 31440253 10.3389/fimmu.2019.01878 PMC6692712

[75] Silva‐Vilches C , Ring S , Mahnke K . ATP and its metabolite adenosine as regulators of dendritic cell activity. Front Immunol. 2018;9:2581. 30473700 10.3389/fimmu.2018.02581 PMC6237882

[76] Schnurr M , Toy T , Shin A , et al. Role of adenosine receptors in regulating chemotaxis and cytokine production of plasmacytoid dendritic cells. Blood. 2004;103:1391‐1397. 14551144 10.1182/blood-2003-06-1959

[77] Ma S , Deng W , Liu J , et al. Blockade of adenosine A2A receptor enhances CD8 T cells response and decreases regulatory T cells in head and neck squamous cell carcinoma. Mol Cancer. 2017;16:99. 28592285 10.1186/s12943-017-0665-0 PMC5461710

[78] Wu D , Sanin D , Everts B , et al. Type 1 interferons induce changes in core metabolism that are critical for immune function. Immunity. 2016;44:1325‐1336. 27332732 10.1016/j.immuni.2016.06.006 PMC5695232

[79] Huang LT , Li X , et al. HIF‐1‐mediated suppression of acyl‐CoA dehydrogenases and fatty acid oxidation is critical for cancer progression. Cell Rep. 2014;8:1930‐1942. 25242319 10.1016/j.celrep.2014.08.028

[80] Su Y , Wu P , Lin S , Huang W , Kuo Y , Lin H . Prognostic value of the overexpression of fatty acid metabolism‐related enzymes in squamous cell carcinoma of the head and neck. Int J Mol Sci. 2020;21. 10.3390/ijms21186851 PMC7559281 32961983

